# The role of XLIF in spinal revision surgery involving failed interbody implants: a review of technique, outcomes, and indications

**DOI:** 10.1007/s00701-025-06643-z

**Published:** 2025-08-13

**Authors:** Yusuf-Zain Ansari, Arwa Jader, Abdul Hameed Kidwai, Dia R. Halalmeh

**Affiliations:** 1 Department of Biology, Temple University, 1801 N Broad St, Philadelphia, PA 19122 USA; 2 Department of Neurosurgery, University of Kufa, Kufa, Iraq; 3 Department of Neurosurgery, University of Iowa Hospital & Clinics, Iowa City, Iowa USA

**Keywords:** Extreme Lateral Interbody Fusion, Lateral Lumbar Interbody Fusion, Foreign Body Removal, Spinal Revision Surgery

## Abstract

**Background:**

This review examines the novel application of the Extreme Lateral Interbody Fusion (XLIF) approach for the removal of foreign bodies in the spine, including subsided cages, failed hardware, and other implants. It aims to evaluate the efficacy, safety, and technical considerations of XLIF in this context and compare it to traditional anterior and posterior surgical approaches.

**Methods:**

A comprehensive literature search in accordance with PRISMA was conducted using Google Scholar, PubMed/MEDLINE, and the Cochrane Library. Search terms included “extreme lateral interbody fusion,” “lateral lumbar interbody fusion,” “cage retrieval,” “revision surgery,” and “foreign body removal.” Articles were selected based on relevance to XLIF use in foreign body removal and included case reports, clinical trials, and observational studies published in English before April 7, 2025.

**Results:**

Seven documented cases met inclusion criteria. The XLIF approach demonstrated advantages such as reduced operative time, blood loss, and shorter hospital stays. It allowed safe access around scar tissue and critical neurovascular structures. The technique was successfully used to remove migrated or failed implants in the lumbar spine with minimal complications, most of which were transient. The approach also enabled the insertion of larger interbody cages, contributing to improved spinal stability and fusion outcomes.

**Conclusion:**

The XLIF approach is a promising alternative for foreign body removal in complex spinal revision surgeries. However, potential complications, such as transient nerve injury, underscore the need for careful patient selection and surgical expertise. Further studies are needed to validate its broader clinical application.

## Introduction

The removal of foreign bodies from the spine can present significant clinical challenges due to the complex surgical corridor and proximity to neurovascular structures. Treatments often require intricate planning to ensure successful outcomes and minimize patient morbidity [2]. Various surgical techniques have been used over the years, with the approach chosen largely dependent on the specific characteristics and location of the foreign body. Among these techniques, the Extreme Lateral Interbody Fusion (XLIF), also known as Lateral Lumbar Interbody Fusion has emerged as a versatile and effective option, particularly in cases where traditional anterior or posterior approaches may be less feasible or carry higher risks.

Despite the increased use of XLIF in revisional surgery, the role of XLIF in removing foreign bodies has not yet been elucidated. The purpose of this comprehensive review of the literature is to examine this particular use-case of XLIF. By reviewing existing literature, we aim to highlight the efficacy, safety, and practical considerations of using XLIF to remove foreign bodies.

## Methods and materials

A thorough literature search was conducted following the Preferred Reported Items for Systematic Review and Meta-analysis (PRISMA) guidelines. The following keywords were used in every possible combination within Google Scholar, Pubmed/Medline, and Cochrane Library: “extreme lateral interbody fusion,” “lateral lumbar interbody fusion,” “cage retrieval,” “revision surgery,” and “foreign body removal”. All the studies were searched before April 7, 2025 and search queries were limited to only display articles written in the English language. We limited the search to English-language articles due to the linguistic capabilities of the review team and the expectation that the majority of relevant surgical literature would be available in English. Duplicate articles were identified and removed from our system.

Eligibility for selection within this review incorporated the following criteria: (1) use of XLIF to remove a foreign object from the spine; (2) case reports, clinical trials, retrospective studies, and prospective studies; and (3) published in the English language. We excluded articles that met the following criteria: (1) not related to foreign body removal, (2) abstracts, letters, or reviews without original data, and (3) studies with no reported outcomes of interest. To minimize subjective bias during screening, two reviewers independently assessed the relevance of each article based on the predefined inclusion and exclusion criteria. Discrepancies were resolved through discussion to ensure consistency and objectivity.

Once eligibility was reassessed by a second reviewer, relevant data was retrieved including (1) study design and year of publication; (2) patient demographics and sample size; (3) characteristics of the foreign bodies removed; (4) details of the XLIF procedure, including surgical technique and instrumentation; and (5) clinical outcomes, including success rates, complications, and recovery times. Out of the initial search results, seven articles met inclusion criteria after screening. The most common reasons for exclusion were: (1) irrelevance to foreign body removal and (2) lack of original patient data.

### Risk of bias assessment

Given that all included studies were case reports or small case series, we performed a risk-of-bias assessment using the CARE (Case Report) guidelines. Two independent reviewers evaluated each study for key components of case report quality, including patient information, clinical findings, diagnostic assessment, therapeutic interventions, and follow-up outcomes. Discrepancies were resolved through discussion.

## Results

### Search results

**Fig. 1:**
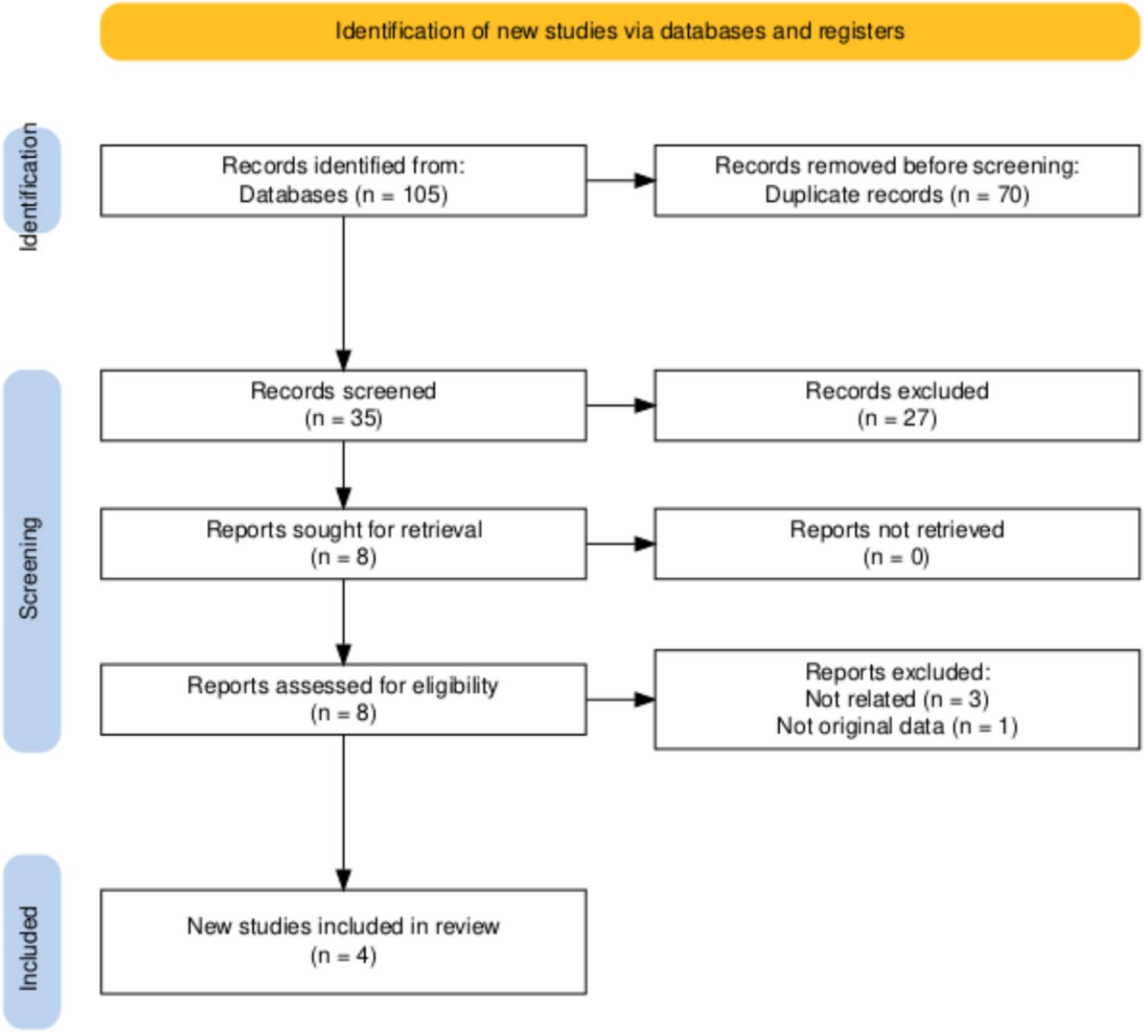
PRISMA diagram

A total of 105 records were identified through database searches. After removing 70 duplicates, 35 unique records were screened. Of these, 8 articles were selected for full-text review. Four articles were excluded due to lack of relevance (*n* = 3) or absence of original data (*n* = 1). Ultimately, 4 studies met the inclusion criteria and were included in this review. The study selection process is summarized in the PRISMA flow diagram (Fig. 1).

### XLIF technique in foreign body removal

The general operative technique for using XLIF for the removal of foreign bodies, such as interbody cages or displaced implants, closely resembles the standard XLIF procedure but with notable deviations to address the complexities of revision surgery. The procedure typically begins with the patient placed in a lateral decubitus position on a radiolucent table, with the iliac crest flexed to maximize exposure to the targeted spinal level [11]. Fluoroscopic guidance is used to accurately localize the disc space or area of interest. A key deviation from the standard XLIF procedure is the extent of exposure and the careful dissection required to navigate around scar tissue and fibrosis from previous surgeries [1, 11]. For instance, Pimenta et al. emphasized the importance of avoiding vascular structures and scar tissue by carefully advancing a split-blade retractor system, such as the MaXcess retractor, to achieve a clear and safe operative field [11]​.

**Fig. 2:**
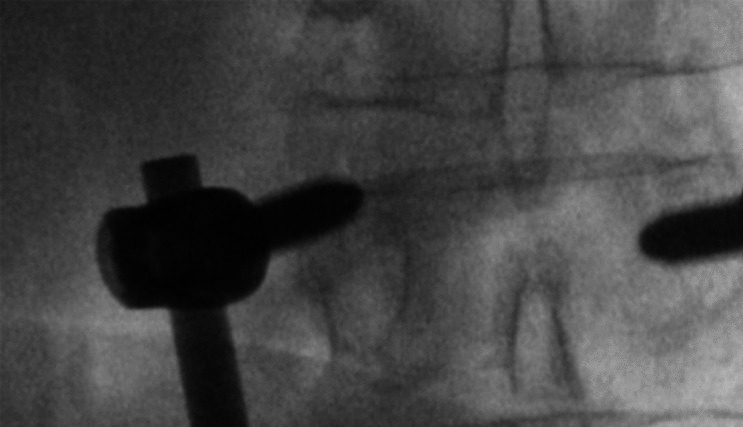
Hook used to retrieve loosened cage Source: Moisi M, Page J, Paulson D, Oskouian RJ (2015) Technical Note – Lateral Approach to the Lumbar Spine for the Removal of Interbody Cages.Cureus 7(5): e268. DOI 10.7759/cureus.268

Once the disc space is accessed, the next step involves the careful loosening of the foreign body, which could be an interbody cage or an artificial disc component. Moisi et al. described the use of an osteotome to gently separate the cage from the endplates and surrounding scar tissue, a step that is crucial to avoid pushing the cage further into the spinal canal or causing nerve damage [8]​. Additionally, depending on the complexity of the case, instruments like curved curettes or hooks may be employed to fully mobilize and retrieve the foreign body as shown in Fig. 2. Eom et al. introduced an innovative technique where a taphole is drilled into the cage, and a thread-tipped stick is used to secure the cage during extraction, preventing further displacement during removal [5].

After the foreign body is removed, the disc space is prepared for fusion by clearing any remaining disc debris and ensuring proper alignment. This preparation is similar to standard XLIF procedures but may require additional steps to manage the altered anatomy due to prior surgeries. In many cases, a larger interbody cage is inserted to provide better stability and support, as noted by Al-Rabiah et al., who highlighted the advantages of using larger cages in revision surgeries to enhance fusion outcomes [1]​. Finally, the closure of the surgical site follows standard procedures, although supplemental fixation, such as percutaneous pedicle screws, may be added to ensure stability, especially in revision cases where the anterior column's integrity is compromised.

### Summary of cases

A total of four studies reporting on the use of XLIF for removal of spinal foreign bodies were included. A summary table shows patient demographics, indications for surgery, type of foreign body removed, and complications (Table 1). The current literature on the use of XLIF for foreign body removal is primarily centered on revision surgeries for failed lumbar interbody fusions.

**Table 1:** Summary of cases involving XLIF for foreign body retrieval

Case	Author, Year	Journal	Age	Type of Foreign Body	Spinal Level(s)	Clinical Presentation	Post-op Complications
1	Pimenta, 2006	Journal of Neurosurgery: Spine	39	Charité Artificial Disc	L4-5	back pain	None
2	Pimenta, 2006	Journal of Neurosurgery: Spine	50	TDR implant	L3-4	scoliosis	Muscle weakness
3	Moisi, 2015	Cureus	71	TLIF interbody cage	L3-4	back pain	None
4	Moisi, 2015	Cureus	52	PLIF interbody cage	L3-4	back pain	None
5	Moisi, 2015	Cureus	51	TLIF interbody cage	L3-4	back pain	None
6	Eom, 2017	Korean Journal of Spine	76	TLIF interbody cage	L4-5	back pain	None
7	Al-Rabiah, 2021	Cureus	42	TLIF interbody cage	L4-5	back pain	None

### Case 1

In 2006, Pimenta et al. documented one of the earliest uses of XLIF for the removal of a displaced Charité Artificial Disc in a 39-year-old female patient who had undergone total disc replacement (TDR) at the L4-5 level due to degenerative disc disease. Postoperatively, the patient experienced persistent back pain due to an unrecognized isthmic pars defect fracture, which led to the instability of the implanted device. Despite an initial attempt to stabilize the spine through posterior fusion and subsequent administration of intravenous antibiotics for a developing infection, the patient’s symptoms persisted. Radiographic imaging revealed failure of the fixation rod, necessitating revision surgery. Pimenta opted for the XLIF approach to avoid the risks associated with anterior and posterior approaches, particularly given the presence of scar tissue and the anatomical challenges of the L4-5 level. The XLIF technique allowed for safe access to the lateral aspect of the spine, where the polyethylene core of the disc was easily grasped and removed, followed by the replacement with a polyetheretherketone cage. The patient experienced no postoperative complications, and her symptoms improved significantly, highlighting the efficacy of XLIF in managing complex revision cases [11].

### Case 2

In the same 2006 study, Pimenta et al. reported a second case involving a 50-year-old female who presented with symptomatic adjacent-level disease following a prior L4-5 fusion. The patient underwent a TDR at the L3-4 level, but postoperative imaging revealed improper positioning of the implant, leading to iatrogenic segmental scoliosis. Despite initial conservative management, including rehabilitation and medical therapy, the patient’s symptoms persisted, prompting the need for revision surgery. Given the risks associated with an anterior approach, including potential vascular injuries, and the challenges posed by posterior approaches due to existing scar tissue, the XLIF technique was chosen. This approach facilitated the safe removal of the improperly positioned disc and subsequent insertion of a larger polyetheretherketone cage, supplemented with posterior percutaneous pedicle screw fixation. The patient’s postoperative course was largely uneventful, with only transient quadriceps weakness that resolved by the third day. The success of this procedure further demonstrated the utility of XLIF in cases requiring complex revisions where traditional approaches may pose significant risks [11].

### Cases 3–5

**Fig. 3:**
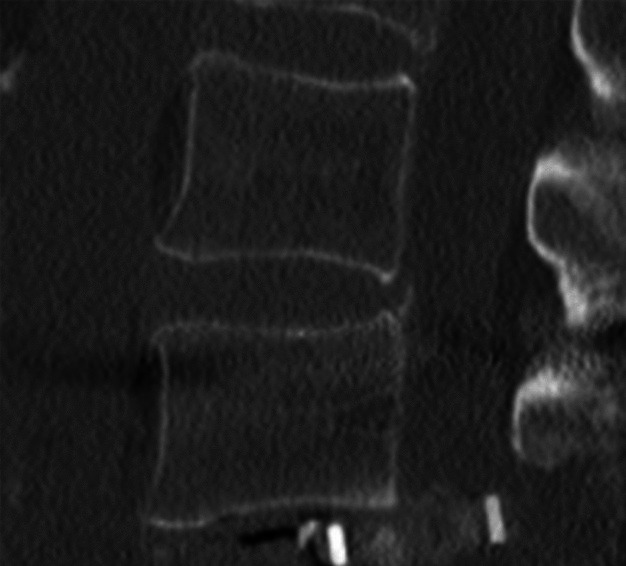
Computed tomography of migrated cage for case 3 Source: Moisi M, Page J, Paulson D, Oskouian RJ (2015) Technical Note – Lateral Approach to the Lumbar Spine for the Removal of Interbody Cages. Cureus 7(5): e268. 10.7759/cureus.268

In 2015, Moisi et al. documented their experience with the XLIF approach for the removal of interbody cages in three patients, all of whom had previously undergone lumbar interbody fusion but subsequently developed complications such as cage migration, fracture, or failure to fuse. The first patient was a 71-year-old female who developed severe lumbar and radicular pain following a fall, which led to the displacement of the interbody cage into the spinal canal, causing central canal stenosis (Fig. 3). The second case involved a 52-year-old female who presented with significant neurogenic claudication and radicular pain due to a fractured interbody cage that had migrated into the spinal canal. The third patient, a 50-year-old female, suffered from increasing back pain and pseudoarthrosis at the L3-4 level with subsidence causing significant lumbar stenosis one year after her initial surgery.

In each of these cases, the lateral approach allowed direct access to the interbody cage while avoiding the complications associated with anterior and posterior approaches, such as scar tissue and epidural fibrosis. The technique involved using an osteotome to loosen the cage from the surrounding bone and scar tissue, followed by retrieval using a hook or pituitary forceps. This method proved effective in all three cases, as the cages were successfully removed without the need for additional posterior hardware revision. The patients experienced resolution of preoperative symptoms and successful fusion with the newly inserted cages [8].

### Case 6

Eom et al. in 2017 presented a case involving a 76-year-old male patient who required revision surgery due to the migration of an interbody cage that had been placed during a previous TLIF for L4-5 stenosis and instability. Postoperatively, the patient experienced persistent radicular symptoms due to the displacement of the cage into the subarticular zone of the spinal canal, which led to nerve compression. Traditional approaches posed significant risks, particularly given the patient’s advanced age and the presence of scar tissue from prior surgeries. Eom and colleagues opted for the XLIF technique for effective cage removal. The XLIF approach was further enhanced by a novel technique that involved creating a taphole in the cage and securing it with a thread-tipped stick, which facilitated its removal from the intervertebral space without further migration. Eom et al. reported some difficulty in removing the cage, especially when there is no spacious passage [5].

### Case 7

Al-Rabiah et al. in 2021 reported on a 42-year-old male patient who underwent revision surgery for a failed TLIF at the L4-5 level. The initial surgery, which included decompression and pedicle screw fixation, was complicated by the medial breach of a pedicle screw, requiring its removal shortly after the procedure. Despite this intervention, the patient continued to experience severe low back pain and bilateral radiculopathy. Imaging revealed pseudarthrosis and the improper positioning of the interbody cage, which necessitated revision surgery. The procedure involved the removal of the unfused cage and its replacement with a larger, more stable cage, followed by supplemental percutaneous pedicle screw fixation. The use of XLIF in this context was particularly advantageous as it reduced the risk of further neurovascular injury and minimized operative time and blood loss. The patient’s recovery was smooth, with significant improvement in symptoms and no major postoperative complications, underscoring the benefits of XLIF in revision spine surgery [1].

### Risk of bias

**Fig. 4:**
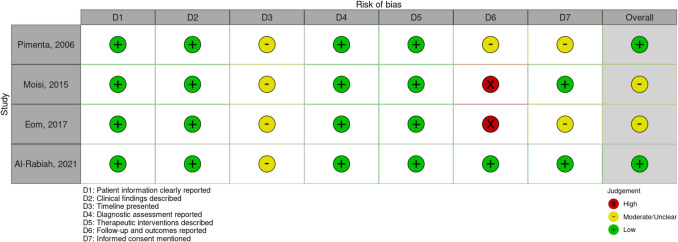
Traffic-light plot

The quality of the included case reports was assessed using seven domains based on the CARE guidelines (Fig. 4). Most studies clearly reported patient information, clinical findings, diagnostic assessments, and surgical interventions. However, common limitations included incomplete reporting of timelines and short or missing follow-up periods. Only two studies explicitly mentioned informed consent.

## Discussion

One of the earliest documented uses of XLIF for foreign body removal was reported by Luiz Pimenta in 2006. In this case, Pimenta utilized the XLIF technique to retrieve a displaced Charité Artificial Disc from a patient who had previously undergone TDR. The choice of XLIF was driven by the need to avoid the risks associated with anterior approaches, such as vascular injury and complications related to scar tissue formation. Vascular injury during anterior approaches is particularly concerning at the L4-5 level where the great vessels must be mobilized. The lateral approach provided access to the spine while aiming to minimize these risks, suggesting the potential of XLIF as an alternative for complex revision surgeries. This case helped introduce XLIF into the discussion of surgical options for such scenarios [11].

While avoidance of vascular injury, a notable concern in anterior approaches, is one of the main advantages of XLIF for foreign body removal, XLIF also minimizes the risks associated with epidural fibrosis and scar tissue that often complicate posterior approaches. Eom et al. emphasized that the lateral approach reduces the likelihood of neurological deficits and incidental dural injuries by circumventing the scarred surgical corridors often encountered in revision surgeries performed via posterior approaches [5]. This is particularly important in cases where reoperation is required, as the natural anatomical planes may have been altered, increasing the risk of nerve damage during surgery.

XLIF offers some other advantages compared to other lumbar interbody fusion techniques such as Transforaminal Lumbar Interbody Fusion (TLIF), Anterior Lumbar Interbody Fusion (ALIF), and Posterior Lumbar Interbody Fusion (PLIF). XLIF has been reported to provide effective interbody stabilization and indirect neural decompression while minimizing major visceral and vascular injuries often associated with ALIF, as well as trauma to paraspinal muscles and facet joints linked to TLIF and PLIF procedures [6, 10]. Additionally, XLIF has been reported to have reduced operative times, blood loss, and shorter hospital stays compared to traditional approaches [1, 4], and it demonstrates superior outcomes in maintaining disc height and segmental angle due to lower stress peaks in the cortical endplate and cancellous bone [7]. Additionally, due to the wider cages used in XLIF, there is a lower risk of cage subsidence as compared to TLIF and PLIF which utilize smaller-sized banana and bullet cages [3, 9].

The use of XLIF for removal of interbody devices carries its own set of potential complications. One of the most commonly reported is the risk of transient nerve injuries due to the proximity of the lumbar plexus [5, 8]. Moisi et al. highlighted the importance of careful navigation through the psoas muscle to avoid nerve injury, which can result in temporary weakness, pain, or numbness in the anterior thigh [8]. These complications often resolve, but their occurrence underscores the importance of intraoperative monitoring. Additionally, Pimenta et al. noted that while the lateral approach avoids direct contact with major vessels, revision surgeries may still pose risks of vascular injury, especially in cases with significant retroperitoneal fibrosis [11]. Eom et al. also described technical challenges such as the risk of implant migration during removal, which could complicate the procedure [5]. Furthermore, Al-Rabiah et al. reported cases where pseudarthrosis and instability following foreign body removal necessitated the placement of larger cages and supplemental fixation to achieve stability [1].

### Limitations

This review has several limitations. The literature on XLIF for spinal foreign body removal is limited to isolated case reports and small series, which lack control groups and introduce publication bias. Quantitative data are sparse and most studies report qualitative outcomes without standardized metrics, making cross-comparison difficult. Follow-up periods are inconsistent, and objective outcome measures such as validated pain scores, functional scales, or fusion assessments are rarely reported. Additionally, the inclusion of English-only articles could introduce language bias. More robust, prospective, and quantitatively rigorous studies are needed to validate the safety, efficacy, and indications of XLIF in this context.

## Conclusion

The application of XLIF for the removal of foreign bodies in spinal revision surgeries has demonstrated significant promise as a safe and effective alternative to traditional anterior and posterior approaches. The lateral approach utilized in XLIF minimizes the risks associated with vascular injury, scar tissue, and epidural fibrosis, which are commonly encountered in other surgical techniques. However, despite these advantages, the procedure is not without its challenges, including potential nerve and vascular complications, which underscore the need for careful patient selection and surgical expertise. As the number of spinal surgeries continues to rise, particularly in an aging population, XLIF may become increasingly recognized as a vital tool in the surgeon's arsenal for managing complex revision cases involving foreign body removal. Future research and clinical experience will further elucidate its role and refine the techniques to maximize patient outcomes.

## Data Availability

No datasets were generated or analysed during the current study.

## Abbreviations

XLIF: Extreme Lateral Interbody Fusion
TDR: Total disc replacement
TLIF: Transforaminal Lumbar Interbody Fusion
ALIF: Anterior Lumbar Interbody Fusion
PLIF: Posterior Lumbar Interbody Fusion

## Declarations

### Registration

Not registered in PROSPERO.
